# Resolution of a periapical lesion in an autoimmune pancreatitis patient treated with long-term low-dose glucocorticoids: a case report

**DOI:** 10.1186/s13005-023-00366-1

**Published:** 2023-06-07

**Authors:** Sohar Flisfisch, Edgar Schäfer

**Affiliations:** 1 University of Bonn, University of Münster, Private Practice, Steinengraben 67, Basel, 4051 Switzerland; 2 Central Interdisciplinary Ambulance in the School of Dentistry, University of Münster, Albert-Schweitzer-Campus 1, building W 30, 48149 Münster, Germany

**Keywords:** Apical periodontitis, Autoimmune pancreatitis (AIP), Glucocorticoids

## Abstract

**Background:**

Patients diagnosed with an autoimmune disease are often systemically medicated with glucocorticoids. Autoimmune pancreatitis (AIP) Type 1 is considered a rare autoimmune disease, which is very well responsive to glucocorticoids and hence can be treated optionally on a long-term basis using low dose of the drug. Apical lesions of root canal-treated teeth can be solved by retreatment of the preexisting root canal obturation or via surgical approaches.

**Case presentation:**

This case report relates to a 76-year-old male patient, whose symptomatic acute apical periodontitis was treated nonsurgically by root canal treatment. However, overtime both roots of tooth 46 were associated with asymptomatic apical lesions. Despite progression of the lesions, due to a painless situation, the patient refrained from proceeding with any further treatment options after explaining the pathological pathway with all its consequences. A few years later the patient was prescribed 2.5 mg glucocorticoid prednisone daily for long-term therapy due to an AIP Type 1. Six years later under strict glucocorticoid therapy the apical lesions healed nearly completely and the patient remained free of symptoms without any further interventions.

**Conclusions:**

These observations suggest that prospective clinical studies are required to further elucidate the potential healing effect of systemic long-term low-dose glucocorticoid medication on lesions of endodontic origin.

## Background

Patients with autoimmune diseases are often confronted with the use of glucocorticoids [1]. The effects of these immune system modulating drugs are versatile as they influence cellular as well as humoral pathways of the immune response, which can be accomplished by genomic or rapid non-genomic expression [2]. Hence, it can be deduced that glucocorticoids have an impact on pathological processes around teeth as well as on periradicular tissues. This is in accordance with a recent animal study showing that glucocorticoids influence the development of experimental apical periodontitis. By causing alteration in the cellular and humoral immune response particularly in polymorphonuclear as well as mononuclear cells and TNF-α, IL-1β and IL-6 secretion, glucocorticoids promote the development of experimental apical periodontitis in rats. As collagen is the main protein degraded during development of apical periodontitis the decrease of collagen type I and III was also observed [3].On the other hand, it has been shown that glucocorticoids may have an inhibitory effect on clastic cells like dentinoclasts or osteoclasts [4].

Primarily failed root canal treatment can be experienced due to different reasons like persistence of bacteria, secondary infection due to coronal or apical leakage, untreated root canals, anatomical complexities (isthmuses and apical ramifications) or instrument fracture. In these cases, formation of intraradicular biofilms may lead to post-treatment apical periodontitis [5], and retreatment or surgical approaches may be considered [6].

Autoimmune pancreatitis (AIP) is a systemic autoimmune disease that can be present along with other autoimmune conditions [7]. AIP is very well responsive to glucocorticoid therapy, and glucocorticoids are therefore the first drugs of choice [8]. With a prevalence of 0.82 per 100’000 in a Japanese population and a yearly incidence rate of 0.29 per 100’000 in a German population, AIP is considered a rare autoimmune disease [9, 10]. AIP is characterized by two appearances. Type 1 is associated with increased levels of immunoglobulin G4 (IgG4) positive plasma cells whereas Type 2 is associated with granulocytic epithelial lesions leading to an idiopathic duct centric pancreatitis [11]. A considerably wide multicenter study revealed that the average age is 61.4 years at first diagnose of AIP Type 1 with 77% affection in male patients [12].

The present case report relates to a patient who developed chronic apical lesions associated with tooth 46 and later was obliged to take lifelong low dose glucocorticoids due to an AIP Type 1, which led subsequently to a nearly complete healing of the apical lesions without any further endodontic interventions.

## Case report

**Fig. 1:**
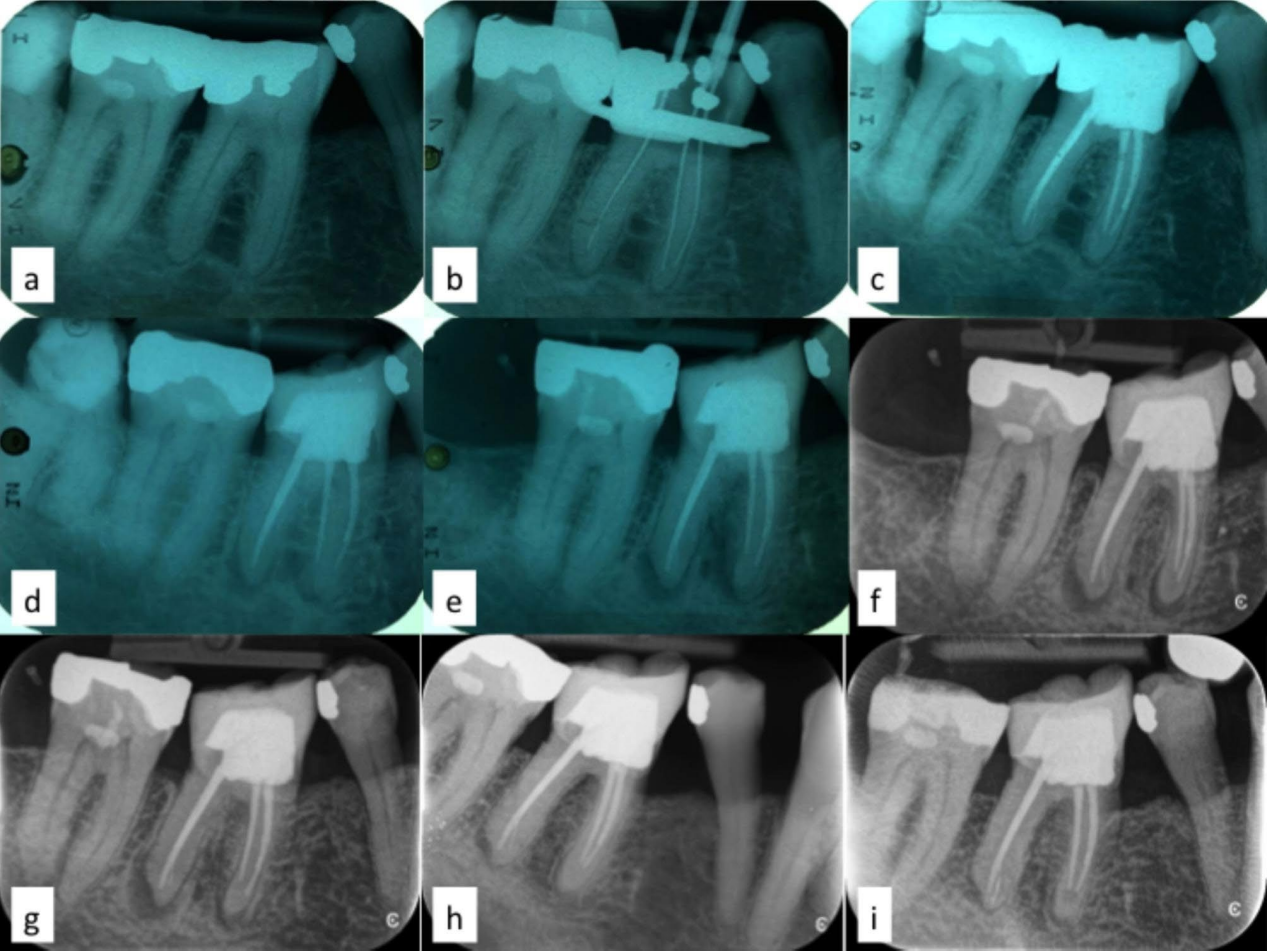
(**a**) Diagnostic periapical radiograph. (**b**) Working length radiograph. The tip of fractured instrument is detectable in the apical portion of the distal root canal. (**c**) Radiograph after root canal filling and core placing. (**d**) One year follow-up periapical radiograph showing lesions around the tips of the mesial and distal root. (**e**) Three years follow-up periapical radiograph showing increase of lesions. (**f**) Five years follow-up periapical radiograph with lesions at both root tips. Beginning of regular systemically lifelong prednisone intake. (**g**) Seven years follow-up periapical radiograph showing decrease of lesions even more pronounced at the mesial root. (**h**) Ten years follow-up periapical radiograph showing almost complete healing of the former lesions. (**i**) Eleven years follow-up periapical radiograph with nearly complete resolution of the former lesions.

A 76-year-old male patient presented with symptoms of an apical periodontitis proceeding from tooth 46. The periapical radiograph of tooth 46 revealed an overhang of an amalgam restauration into the distal interspace and a slight widening of the periodontal ligament was evident (Fig. 1a). Pulp sensibility testing (-78 °C) was negative while the occlusal percussion test was positive. The patient gave informed consent to root canal treatment of the affected tooth. The treatment was performed in three appointments. Following application of rubber dam, access cavity preparation and localization of the root canal orifices, an apex locator (Propex, Dentsply Sirona, Bensheim, Germany) was used for determination of the working lengths. Instrumentation of the canals was performed with both hand nickel-titanium (NiTi) files and engine-driven Hero Shaper (Micro Mega, Besançon, France) up to size 30 and 4% taper. All canals were extensively rinsed with a 2% sodium hypochlorite solution. However, during the first appointment an engine-driven instrument fractured in the apical portion of the distal root, which could not be removed (Fig. 1b). The patient was immediately informed about this circumstance and possible consequences. After the first and second appointment, calcium hydroxide was used as an intracanal dressing, and the access cavity was sealed with an intermediate restorative material. The root canal filling with gutta-percha and AH Plus (Dentsply Sirona, Bensheim, Germany) sealer was established according to the cold lateral compaction technique two weeks after the beginning of the treatment, while the patient was free of symptoms. The access cavity was sealed with an adhesively bonded composite (Tetric, Ivoclar Vivadent, Glattpark, Switzerland) core. The root canal fillings in both mesial root canals and distal root canal were short (about 1.5 mm from the apex in the distal root and about 3 mm from the apex in the mesial root) due to substantial intracanal calcification (Fig. 1c).

As the patient stayed free of symptoms, a full ceramic crown was inserted two months later to guarantee coronal sealing and stabilization of the tooth. One year later, the follow-up radiograph revealed slight translucencies around both root tips. The patient was elucidated about this finding and adequate treatment plans for solving this issue were explained (Fig. 1d). However, the patient refrained from further treatment options as he was free of symptoms and due to his age, he was not willing to put any further efforts in an asymptomatic tooth. Consequently, two years later the apical lesions increased significantly in size, which was documented radiographically and possibilities to address this issue were again discussed with the patient (Fig. 1e). But the patient still was not interested in any treatment option as he was still free of symptoms.

Another two years later, in total five years after root canal obturation, the patient was diagnosed with an AIP type 1, which was treated with prednisone 10 mg per day for one month and then on a low dose basis of 2.5 mg per day for long-term therapy (Table 1). At this time the periapical lesions associated with booth roots remained nearly unchanged (Fig. 1f). Another two years later and still under the maintenance dosage of 2.5 mg of prednisone per day, the periapical lesions decreased in size, but even more at the mesial root (Fig. 1g). Three additional years later, which was in total 10 years after the root canal treatment and 5 years after beginning of glucocorticoid therapy, both lesions disappeared almost completely (Fig. 1h). Another year later (11 years after root canal treatment and 6 years after beginning of glucocorticoid therapy) the former lesions healed nearly completely (Fig. 1i). The patient is still instructed by his internist to continue lifelong with the glucocorticoid therapy taking 2.5 mg prednisone per day.

**Table 1:** Summary of the medical history and medications of the patient

Date	Medication		Anamnesis
1979–2014	none		Non-smoker Inconspicuous medical history
2014–2015	Allopurinol		Gout
May 2016	Glucocorticoid prednisone from 10 mg for one month to 2.5 mg glucocorticoid prednisone daily		Autoimmune pancreatitis (AIP) Type 1
August.2016	2.5 mg glucocorticoid prednisone daily (until today)		Coloscopy Ectomy of adenoids
2021	Atorvastatin (until today)		High LDL level
2023	3 g Metamizole per day for two weeks	Femoral neck fracture	

## Discussion

Conventional orthograde retreatment of a root canal-treated tooth with a persistent or progressive apical lesion should be the first treatment option of choice [13]. Even though retreatment has a success rate of two third of the cases [14] apical surgery might be considered in some cases [15]. Especially in the present case where not only the full length of the root canals could not be explored but additionally an instrument fractured in the most apical part of the distal root canal, apical surgery might address both issues and would preserve the coronal restauration. However, it should be considered that the prognosis of root canal treatment with a fragment left in the root canal is not significantly decreased per se [16].

As AIP Type 1 is a rare disease, most studies suffer from relatively low patient numbers especially for long-term therapy results [17]. One of the challenges in the treatment concept of AIP Type 1 is the dosage of glucocorticoids, which is the treatment of choice in most AIP Type 1 cases these days for long-term therapy preventing relapse [18, 19]. From international consensus for the treatment of AIP it is known that some patients may profit from a long-term glucocorticoid therapy with AIP Type 1 [20].

The potential effect of glucocorticoids has been described in several studies with diverse effects. In vitro studies have shown that glucocorticoids not only promote the proliferation of human dental pulp cells but can also initiate the mineralization of these cells and hence have the potential to repair injured pulp tissues by down-regulating the expression of pro-inflammation-related genes and up-regulating the expression of anti-inflammatory genes [21, 22]. Thereby, a sustained release of the glucocorticoid seems to efficiently enhance odontogenic differentiation in vitro [23]. Furthermore, it has been shown that the mineralization of bone can be initiated by the use of glucocorticoids in mature osteoblasts [24]. In animal studies a decrease of bone mineralization was observed under glucocorticoid administration and interestingly glucocorticoids showed the potential to inhibit the recruitment of bone-resorbing cells and stimulate the activity of existing osteoclastic cells independently [25, 26]. A study in rats showed that systemic glucocorticoid medication at a dose equivalent to a short-term high dose used in humans significantly inhibited the growth of periapical lesions. The authors speculated that (i) bone resorption in periapical inflammatory lesions may be pharmacologically down regulated by glucocorticoids due to inhibition of the production of inflammatory cytokines by lipopolysaccharide-activated macrophages at the transcriptional level and (ii) that glucocorticoids may cause enhanced apoptosis of osteoclasts [27]. However, a more recent animal study with a similar design but higher concentrations of glucocorticoids suggests that the inhibitory effect of glucocorticoids on periapical pathosis might be dose dependent as the effect of glucocorticoids was contrary in this study [3].

In a clinical study a correlation between high dose application of glucocorticoid and extensive narrowing of the dental pulp chamber due to distinct formation of secondary dentin in patients with renal transplantation and systemic corticosteroids medication was described [28]. Yet, all the mechanisms are still not fully understood.

The present case report describes for the first time the long-term effect of low-dose glucocorticoid prednisone on a before progressive periapical lesion, which healed nearly completely. Possibly, one could object that the nearly complete healing had no causal connection to the glucocorticoid intake, but was due to a spontaneous healing. However, spontaneous healing of apical lesions is unlikely in this case due to the continuing improvement of the apical lesions from the start of therapy with glucocorticoid intake. Moreover, spontaneous healing of the apical region after development of a progressive lesion has not been described in the literature yet. Only a case of remission of an apical lesion after orthodontic movement of the affected tooth has been described so far [29]. Thus, against this background the resolution of the apical periodontitis seems to mainly be affected by the long-term low-dose glucocorticoid intake (Table 1). After 10 years of the root canal treatment the patient was prescribed another medication, namely the statin Atorvastine (Table 1). Although an association between long-term (2 years and longer) statin intake and healing of apical periodontitis has been demonstrated in a cohort study [30], a possible additive effect of the statin intake of the patient on the resolution of the apical periodontitis can be precluded as the timespan between initiation of the statin intake and the last follow-up radiograph showing nearly complete resolution of the former lesions was less than one year (Fig. 1i). Albeit, a statin intake of at least 2 years is required to expect any beneficial effect on the healing of periapical pathosis [30].

Limitations of the present case report are twofold. Firstly, radiographs over the observation period of 11 years have not been standardized in terms of angulation. Secondly, cone-beam computed tomography has not been used to verify the three-dimensional extent of the periapical lesion and to ensure that the lesions have healed nearly completely.

Systemic lupus erythematosus (SLE) is another, more common autoimmune disease where it has been shown that the administration of glucocorticoids had an adverse effect on the periapical tissues. In a study among patients treated for SLE the usage of glucocorticoids resulted in a higher prevalence of periapical abscesses [31]. However, it should be taken into consideration that the treatment of SLE requires a high dose of glucocorticoids, which is associated with more side effects per se [32]. Moreover, the total dosage of glucocorticoids administrated for therapeutic purposes has an impact on possible side effects and should be therefore distinguished between high dose (˃15 mg/d) and low dose (˂7.5 mg/d) application [33]. Hence, the observations of this case report suggest that prospective clinical studies are required to further elucidate the potential healing effect of systemic long-term low-dose glucocorticoid medication on lesions of endodontic origin.

## Conclusions

Within the limitations of this case report, the potential benefits of low-dose glucocorticoids in a long-term prescription might influence the healing potential of the periapical region beneficially. Patients with long-term low-dose glucocorticoids intake should be screened for asymptomatic apical lesions and, in case of impossibility of traditional treatment options, should be monitored after glucocorticoid medication on a regular basis radiographically.

## Data Availability

Not applicable.
